# Tracking the Spread of the BA.2.86 Lineage in Italy Through Wastewater Analysis

**DOI:** 10.1007/s12560-024-09607-1

**Published:** 2024-06-25

**Authors:** C. Veneri, D. Brandtner, P. Mancini, G. Bonanno Ferraro, M. Iaconelli, E. Suffredini, M. Petrillo, G. Leoni, V. Paracchini, B. M. Gawlik, A. Marchini, the SARI Network, Paolo Torlontano, Giuseppe Aprea, Silvia Scattolini, Vicdalia Aniela Acciari, Michele La Bianca, Rosa Anna Cifarelli, Achille Palma, Giuseppe Lauria, Giovanna La Vecchia, Vincenzo Giordano, Luigi Cossentino, Francesca Pennino, Annalisa Lombardi, Lisa Gentili, Paola Angelini, Daniele Nasci, Giovanni Alborali, Nicoletta Formenti, Flavia Guarneri, Nadia Fontani, Marco Guercio, Marika Mariuz, Gabriella Trani, Anna Pariani, Laura De Lellis, Carla Ancona, Alessandra Barca, Flavia Serio, Doriana Antonella Giorgi, Irene Ferrante, Valeria Capparuccini, Maria Teresa Scicluna, Antonella Cersini, Gabriele Pietrella, Elena Nicosia, Nadia Fontani, Marco Guercio, Elena Grasselli, Alberto Izzotti, Irene Tomesani, Marta Bellisomi, Stefano Rosatto, Emanuela Ammoni, Danilo Cereda, Barbara Bertasi, Marina Nadia Losio, Desdemona Oliva, Sara Castiglioni, Silvia Schiarea, Sandro Binda, Valeria Primache, Laura Pellegrinelli, Clementina Cocuzza, Rosario Musumeci, Luigi Bolognini, Fabio Filippetti, Marta Paniccia’, Sara Briscolini, Silvia Magi, Annalisa Grucci, Michele Colitti, Angela Ciccaglione, Carmen Montanaro, Bartolomeo Griglio, Angela Costa, Lucia Decastelli, Angelo Romano, Manila Bianchi, Elisabetta Carraro, Cristina Pignata, Manuela Macrì, Silvia Bonetta, Nehludoff Albano, Giuseppe Di Vittorio, Onofrio Mongelli, Francesca Apollonio, Francesco Triggiano, Osvalda De Giglio, Maria Teresa Montagna, Mario Palermo, Carmelo Massimo Maida, Walter Mazzucco, Fabio Tramuto, Simona De Grazia, Giovanni Maurizio Giammanco, Chiara Filizzolo, Giuseppa Purpari, Francesca Gucciardi, Margherita Ferrante, Antonella Agodi, Martina Barchitta, Piergiuseppe Cala’, Annalaura Carducci, Marco Verani, Ileana Federigi, Salvatore Macrì, Ermanno Federici, Maya Petricciuolo, Agnese Carnevali, Francesca Russo, Gisella Pitter, Vanessa Groppi, Franco Rigoli, Marco Zampini, Tatjana Baldovin, Irene Amoruso, Maria Cadonna, Mattia Postinghel, Paola Foladori, Lorella Zago, Alberta Stenico, Morelli Marco, Dossena Matteo, G. La Rosa

**Affiliations:** 1 National Center for Water Safety (CeNSiA), Istituto Superiore di Sanità, Viale Regina Elena 299, 00161 Rome, Italy; 2 Department of Infectious Diseases, Istituto Superiore di Sanità, Rome, Italy; 3 Department of Food Safety, Nutrition and Veterinary Public Health, Istituto Superiore di Sanità, Rome, Italy; 4 Seidor Italy S.r.l., 20129 Milan, Italy; 5 European Commission, Joint Research Centre (JRC), Ispra, Italy; 6 European Commission, Joint Research Centre (JRC), Geel, Belgium

**Keywords:** SARS-CoV-2, BA.2.86, JN.1, Wastewater surveillance, Digital PCR

## Abstract

The emergence of new SARS-CoV-2 variants poses challenges to global surveillance efforts, necessitating swift actions in their detection, evaluation, and management. Among the most recent variants, Omicron BA.2.86 and its sub-lineages have gained attention due to their potential immune evasion properties. This study describes the development of a digital PCR assay for the rapid detection of BA.2.86 and its descendant lineages, in wastewater samples. By using this assay, we analyzed wastewater samples collected in Italy from September 2023 to January 2024. Our analysis revealed the presence of BA.2.86 lineages already in October 2023 with a minimal detection rate of 2% which then rapidly increased, becoming dominant by January 2024, accounting for a prevalence of 62%. The findings emphasize the significance of wastewater-based surveillance in tracking emerging variants and underscore the efficacy of targeted digital PCR assays for environmental monitoring.

**Supplementary Information:**

The online version contains supplementary material available at 10.1007/s12560-024-09607-1.

## Introduction

The emergence of new variants of SARS-CoV-2 remains a dynamic and ongoing phenomenon. It is important to keep abreast of new variants that may arise, through the development or targeted detection assays. Following the declaration of the end of the pandemic, surveillance efforts are limited due to a decline in clinical testing and sequencing (Attar Cohen et al., 2023). This presents a significant challenge in tracking the insurgence of new variants. By the end of 2023, the SARS-CoV-2 XBB lineages, mainly EG.5.1, predominated globally. However, in July 2023, a lineage very different from XBB emerged (World Health Organization (WHO), 2023), named BA.2.86. First detected in Denmark and Israel, it subsequently appeared in multiple regions worldwide. In Italy, BA.2.86 first appeared in September 2023 (Caccuri er al., 2023). At the time of writing, BA.2.86 consists of several different sub-lineages, namely BA.2.86.1 to BA.2.86.4, JQ.1, and JN.1- to JN.10 and is referred as BA.2.86* (BA.2.86X) lineage. By January 19, 2024, BA.2.86* lineage had become the dominant lineage in EU/EEA countries (European Centre for Disease Prevention and Control (ECDC), 2024a; European Centre for Disease Prevention and Control (ECDC), 2024b). Due to its high number of spike protein mutations, Omicron BA.2.86 was designated as a Variant Under Monitoring (VUM) on 17 August 2023, predicted to potentially evade vaccine-induced immunity against SARS-CoV-2 infection. Subsequently, in November 2023, the BA.2.86 variant was classified as a Variant Of Interest (VOI) (World Health Organization (WHO), 2023). A significant proportion of BA.2.86* sequences are attributed to the JN.1 sub-lineage, first detected in August 2023, which contains mutations of BA.2.86 + S:L455S. On December 19, 2023, the World Health Organization classified JN.1 as a separate VOI from the parent BA.2.86 lineage due to its rapidly increasing proportion (World Health Organization (WHO), 2023). As on 15 March 2024 JN.1, was the most reported variant of interest globally, accounting for 90.3% of sequences in week 9, while in the same week its parent lineage BA.2.86 was declining and accounted for 2.2% of sequences (World Health Organization (WHO), 2024). According to the Global Initiative on Sharing All Influenza Data (GISAID), 111,325 sequences with the BA.2.86* lineage have been detected in at least 110 countries and 54 US states since the lineage was identified (GISAID—Lineage Comparison). Of these, 93,523 (84%) were associated with JN.1.

The use of wastewater monitoring can provide a comprehensive approach to tracking the circulation and spread of different SARS-CoV-2 variants within a community or population (Bartel et al., 2024; Combe et al., 2024; Cutrupi et al., 2022; Espinosa-Gongora et al., 2023; Lipponen et al., 2024). This method provides a broader perspective beyond individual testing, offering insights into the overall viral landscape and aiding in the early detection of emerging variants (Sapoval et al., 2023).

Beginning in August 2023 and in the following months, BA.2.86* was detected in wastewater samples from several countries, including Sweden, France, Denmark, Germany and Thailand (Espinosa-Gongora et al., 2023; Wurtzer et al., 2024; Bartel et al., 2024; Rasmussen et al., 2023; Wannigama et al., 2023).

The aim of this study was to investigate the occurrence of BA.2.86* in wastewater throughout Italy in the period spanning from October 2023 to January 2024. Following the recommendation of the European Commission (Commission Recommendation (EU) 2021/472 of 17 March 2021 [https://eur-lex.europa.eu/eli/reco/2021/472]), regular environmental surveillance activities for SARS-CoV-2 variants have been carried out at the national level in the period October 2021–March 2023, through monthly monitoring (so called “flash surveys”). The reports are published regularly on the Istituto Superiore di Sanità (ISS) website dedicated to environmental monitoring of COVID-19 (https://www.iss.it/cov19-acque-reflue). Although the official surveillance programme ended in March 2023 due to the cessation of funding, voluntary testing continued. However, some regions reduced the frequency or number of sampling sites. In addition, due to cost considerations, there has been a shift from next-generation sequencing (NGS) of a long fragment of the spike protein to Sanger sequencing, which has limitations in identifying minority variants present in a sample with a mixture of variants. To address this issue, a digital RT-PCR assay (RT-dPCR) was developed to specifically detect BA.2.86*. By using this assay, we investigated when and where the BA.2.86* lineage was introduced in Italy and how it spread over time.

## Material and Methods

### Wastewater Sampling

A total of 507 samples collected between September 2023 and January 2024 were analysed in this study. The samples were part of the wastewater sample collection of the Italian SARI network for wastewater surveillance, implemented following to the Commission Recommendation 2021/472 and Legislative Decree (Decree of the Ministry of Health 30.10.2021, GU Serie Generale n.294 del 11-12-2021 [https://www.gazzettaufficiale.it/eli/gu/2021/12/11/294/sg/pdf]). Samples were collected as a 24-h composite and stored at 4 °C until analysis, which was performed within 48 h of collection. The list of samples is provided in Table S1. In particular, 111 samples were collected in September 2023 (in the week starting September, 4th), 110 samples in October 2023 (in the week starting October, 2nd), 97 in November 2023 (in the week starting November, 6th), 98 in December 2023 (in the week starting December, 1st) and 91 in January 2024 (in the week starting January, 8th). In total, 16 Regions and 2 Autonomous Provinces (A.P.) were involved in the study, with a total of 107 wastewater treatment plants monitored.

### Viral Concentration, Nucleic Acids Extraction and SARS-CoV-2 Quantification by RT-qPCR

The SARI network laboratories managed the sampling process, viral concentration, and nucleic acid extraction using a standardized national protocol as previously described (La Rosa et al., 2021, 2022). Briefly, after heat inactivation at 56 °C for 30 min, 45 mL of the samples were concentrated using a polyethylene glycol (PEG)-based method (Wu et al., 2020). Centrifugation at 4500 × g for 30 min removed larger particles and debris. Subsequently the supernatant (40 mL) was mixed with 8% polyethylene glycol 8000 and 0.3 M NaCl. The mixture was then centrifuged at 12,000 × g for 2 h. The resulting pellet was resuspended in 2 mL of NucliSENS Lysis Buffer reagent for RNA extraction using magnetic silica beads. The eluted RNA (100 μL) was further purified with the OneStep PCR Inhibitor Removal Kit and stored at − 80 °C until molecular analysis. Purified RNAs were sent to the ISS for variant detection as part of the regular flash surveys undertaken since October 2021. The RT-qPCR assay for SARS-CoV-2 was also performed by each laboratory of the SARI network according to previously published protocols (La Rosa et al., 2021, 2022). Quality assurance controls (process control virus, inhibition control) were included to assess viral recovery and PCR inhibition, as previously described (La Rosa et al., 2021). After real-time PCR, the nucleic acids were stored at − 80 °C for further testing and shipped to ISS on dry ice for flash surveys on variants.

### Assay for BA.2.86 Lineage Quantification by Digital RT-PCR

A specific assay was developed using Primer3Plus software (https://www.bioinformatics.nl/cgi-bin/primer3plus/primer3plus.cgi) to selectively quantify the RNA levels of the BA.2.86 lineage of SARS-CoV-2. The assay targets the spike region, spanning from amino acid residue V445H to Y505H, with the probe binding to the region containing the characteristic Val deletion 483 (V483del), together with mutations N481K and E484K, which are specific to the BA.2.86 lineage. Primers and probes used in the present study are shown in Table 1.

**Table 1:** Primer and probes designed in the present study

PCR ID	Primer/probe ID	Nucleotide sequence (5${\prime}$-3${\prime}$)	Orientation	Amplicon size (bp)	Position*	Targeted mutations
1032	Primer Fw—2532	CAAGCTTGATTCTAAGCATAGTGG	+	188	1329–1352	V445H, G446S, N450D, L452W, N460K, S477N, T478K, N481K, V483del, E484K, F486P, Q498R, N501Y
	Primer Rev—2534	CACCATAAGTGGGTCGGAAA	–		1437–1464	
	Probe—2533	HEX-CCGGTAACAAACCTTGTAAAGGTAA-BHQ-1	+		1500–1519	

*Position related to GISAID sequence accession ID: EPI_ISL_18114953, spike protein

To validate the BA.2.86 lineage assay for digital RT-PCR, the limit of detection (LOD) and the limit of quantification (LOQ)were calculated. To determine these parameters, four tenfold dilutions of a BA.2.86 control were tested in three independent runs as triplicates under the same conditions using RT-dPCR (Doğantürk et al., 2023). To evaluate the performance of the assay in wastewater samples, the BA.2.86 control was diluted in nucleic acids extracted from a wastewater sample collected in 2021, long before the circulation of the BA.2.86 variant. LOD_95%_ was calculated according to Wilrich and Wilrich (2009), using the tools available at https://www.wiwiss.fu-berlin.de/fachbereich/vwl/iso/ehemalige/wilrich/index.html. LOQ was calculated as the lowest standard concentration that could be quantified with a CV value below 35% (Klymus et al., 2020).

Digital RT-PCR (RT-dPCR) analysis was conducted using the QIAcuity One 5-plex dPCR system (Qiagen, Hilden, Germany), along with the QIAcuity OneStep Advanced Probe Kit (Qiagen, Hilden, Germany). Each reaction consisted of 12 μL of reaction mixture per well containing 3 μL of Qiacuity 4 × OneStep Advanced Probe Master Mix, 0.12 μL of 100 × OneStep Advanced RT Mix (Reverse Transcription), 0.4 μM of primer forward (ID 2532, CAAGCTTGATTCTAAGCATAGTGG), 0.4 μM of primer reverse (ID 2534, CACCATAAGTGGGTCGGAAA), 0.2 μM of probe (ID 2533, HEX-CCGGTAACAAACCTTGTAAAGGTAA-BHQ-1), and 2.48 of RNase-Free water. Finally, 4 μL of extracted RNA was added as template. For each sample, two technical replicates were performed. Nucleic acids were amplified under the following conditions: reverse transcription for 40 min at 50 °C, enzyme activation for 2 min at 95 °C and 40 cycles of 5 s at 95 °C and 30 s at 60 °C. Partitions were imaged with 700 ms (HEX) exposure time, with gain set to 8. The QIAcuity Software Suite version 2.2.0.26 was used to determine sample thresholds using positive and no-template control wells (NTCs) with the manual global threshold approach. Samples were considered positive if they exhibited at least three positive partitions in one of the two replicates. The target concentration (copies/μl) in each sample was calculated using the instrument result and the formula:

Omicron BA.2.86* (g.c./L) = [dPCR result (copies/μl) × reaction volume/volume of tested RNA] × 100 × 25.

Where 100 is the total volume of extracted RNA and 25 is the ratio between the processed wastewater volume (40 mL) and the reference volume (1 L). Moreover, BA.2.86* viral loads were normalized for both the flow rate and the population equivalents of WWTP (genome copies/day*inhabitant).

Before testing environmental samples, the assay was tested on a clinical sample characterized as BA.2.86, used as a positive control, and, to asses specificity, on three clinical samples of different Omicron variants, namely XBB.1.5, XBB.1.16, and EG.5 (kindly provided by the Department of Infectious Diseases at the Istituto Superiore di Sanità in Rome). In parallel, the same controls were tested with the generic assay for SARS-CoV-2 (La Rosa et al., 2021), adapted for dPCR in this study.

A subset of wastewater samples (n = 28, including 5 samples from December 2023 and 23 from January 2024) was also amplified by conventional RT-PCR using the newly developed BA.2.86 primers and were subjected to Sanger sequencing to further confirm assay specificity and discriminate the JN.1 from the parental BA.2.86 variant. JN.1 differs from the parental variant for its L455S mutation, which falls within the fragment amplified by the newly designed assay.

## Results

The generic SARS-CoV-2 test produced positive results on all clinical samples used as controls. Conversely, the specific test designed for BA.2.86 provided amplification on the BA.2.86 control, but did not detect the variants XBB.1.5, XBB.1.16, and EG.5, demonstrating its specificity for the BA.2.86 lineage. LOD_95%_ calculated according to Wilrich and Wilrich (2009) was 1.82 copies/μL. The coefficient of determination (R^2^) value was calculated to be 0.9944, correlating with the expected concentrations. Therefore the lowest standard concentration that could be quantified with a CV value below 35% was dilution 3, corresponding to a LOQ of 9.16 copies/μL.

The generic assay detected SARS-CoV-2 in 447 out of 507 (88.2%) wastewater samples. The viral loads ranged from 2.5 × 10^2^ to 8.4 × 10^6^ (median value: 1.6 × 10^4^) genome copies (g.c.)/L of wastewater (Supplementary Table 1). The concentration and extraction procedure had an average recovery of 25% (range 0.5–100%) as evaluated by seeding the samples with mengovirus or murine norovirus. The samples’ inhibition, assessed by RT-qPCR as ΔCq from a non-inhibited reference reaction, ranged from zero to 5.1 (median value: 0.17).

Out of 507 analysed samples, 116 (23%) tested positive for the BA.2.86 lineage, with viral concentrations ranging from 4.0 × 10^3^ to 1.1 × 10^5^ genome copies/L (g.c./L), as reported in Table 2.

**Table 2:** RT-dPCR (genome copies/L) results

Sampling month	N° of samples	N° of positive samples	% of positivity	Concentration in positive samples (g.c./L)		
				Geometric mean	Min	Max
September 2023	111	0	0	–	–	–
October 2023	110	2	2	1.2E + 04	4.0E + 03	2.0E + 04
November 2023	97	6	6	1.0E + 04	5.3E + 03	1.7E + 04
December 2023	98	52	53	2.2E + 04	4.0E + 03	1.1E + 05
January 2024	91	56	62	1.8E + 04	4.1E + 03	5.5E + 04
Total	507	116	23	1.9E + 04	4.0E + 03	1.1E + 05

**Fig. 1:**
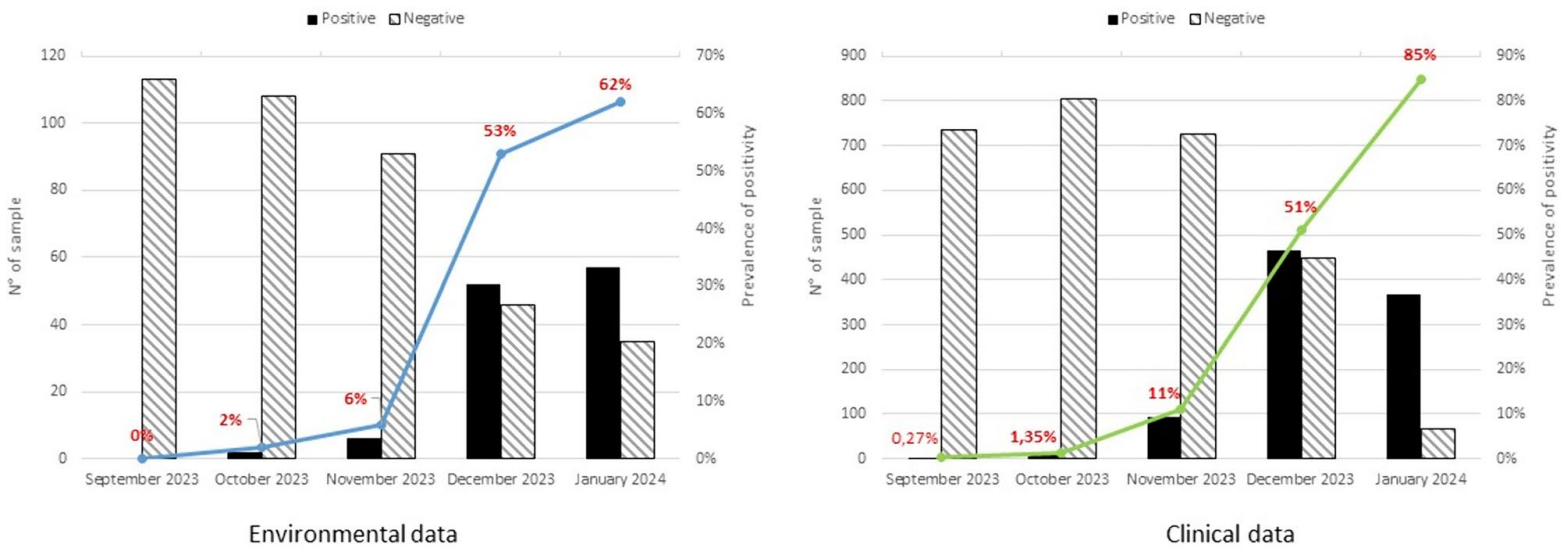
Prevalence and trend of positivity for BA.2.86* over the study period for both environmental and clinical data

**Fig. 2:**
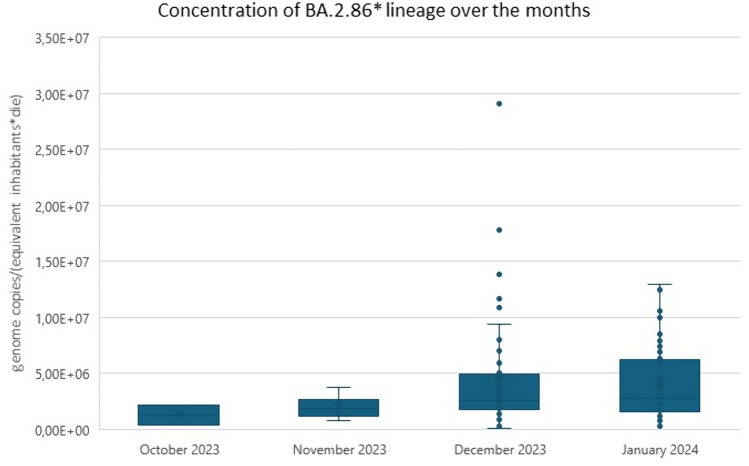
Box plot illustrating the distribution of BA.2.86 lineage normalized concentrations in positive samples across months. Dots indicate individual measurements; the lines of the box represent the 25th, 50th and 75th percentile (bottom, middle, and top lines, respectively); whiskers show the range from minimum to maximum values, not including outliers

The Omicron BA.2.86* was first detected in wastewater in Italy on 2 October 2023, in two samples collected from Pomezia (Lazio) and Trento (A.P. Trento). Figure 1 displays the number of positive and negative samples, as well as the increase in detection rate for the BA.2.86 lineage over time. During the analysed months, the variant rapidly spread: 2% and two Regions/A. P. in the week of 2–6 October 2023; 6% and 5 Regions/A. P. in the week of 6–10 November 2023; 53% and 14 Regions/A. P. in the week of 1–7 December 2023; and 62% and 13 Regions/A. P. in the week of 8–12 January 2024. The BA.2.86 variant was not detectable in any of the September samples analyses.The median BA.2.86 viral loads, normalized for flow rate and equivalent inhabitants, were slightly higher in December and January, as shown in the box plot in Fig. 2. Supplementary Table 1 summarises the BA.2.86* variant concentration in each sample.

**Fig. 3:**
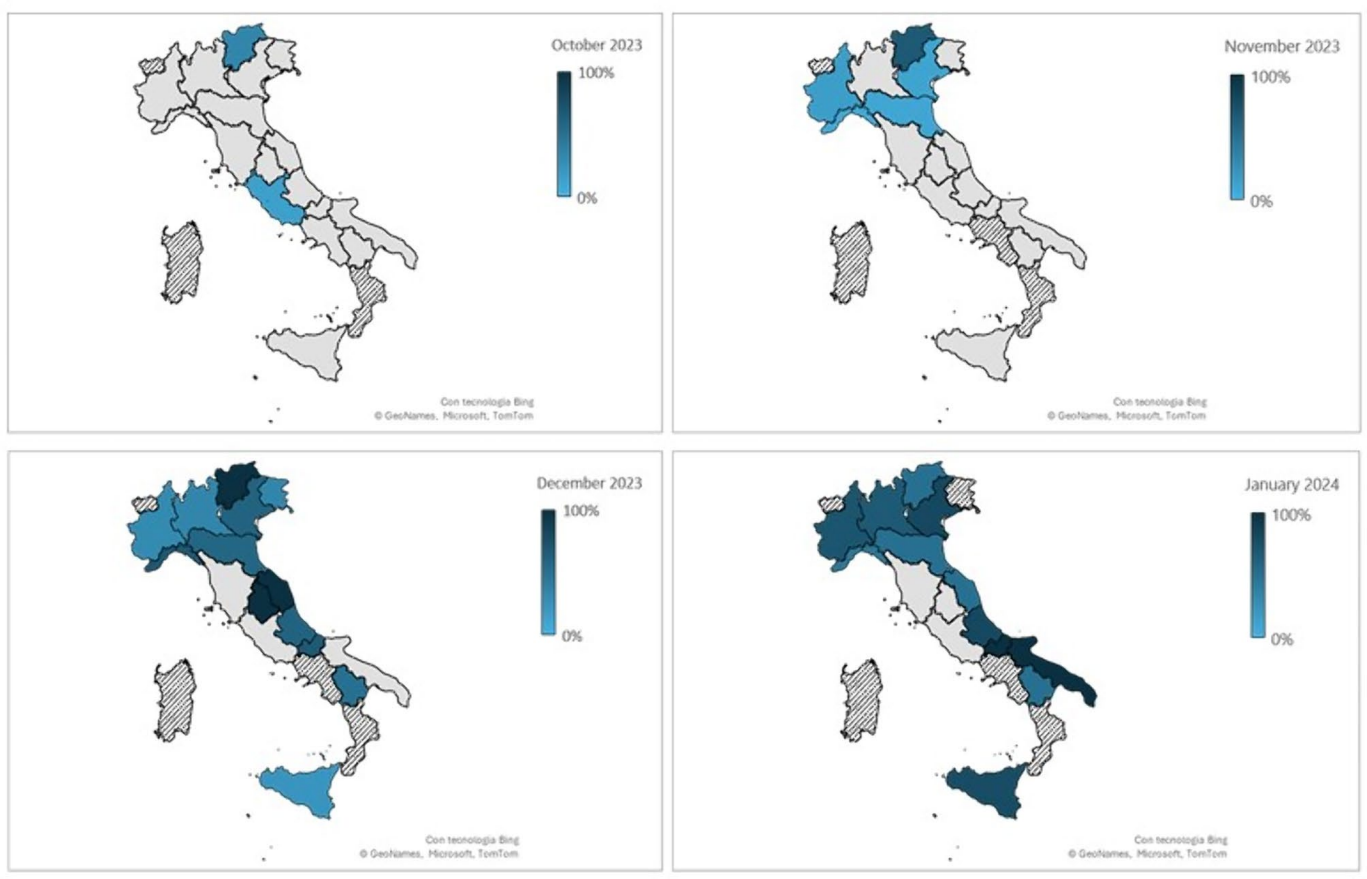
Geographical distribution of BA.2.86 lineage prevalence over four months. The detection of the Omicron BA.2.86* is represented with increasing shades of blue based on its prevalence. Grey Regions/A.P. tested negative for the BA.2.86* variant. Regions/A.P. without any tested sample in a given month are shown in stripes

The occurrence of the Omicron BA.2.86* has progressively increased in most regions over the four months under monitoring, with some exceptions in regions with low sample numbers. Figure 3 provides a detailed geographic distribution and spread of the variant, including the increase in the number of regions where it was detected each month, and the detection rate in each region/A. P. over time.

All 28 subset samples were successfully sequenced and characterised as a JN.1 variant, identified by the key mutation L455S.

## Discussion

Numerous studies worldwide have demonstrated the feasibility of monitoring SARS-CoV-2 variants in wastewater (La Rosa et al., 2023; Rector et al., 2023) and more in general, its utility to monitor evolution and spread of endemic viruses (Yousif et al., 2023). This highlights the crucial role of wastewater surveillance as an alternative strategy for detecting viral variants circulating within communities (Bonanno Ferraro et al., 2022; Cutrupi et al., 2022; Heijnen et al., 2021; Hokajärvi et al., 2021; Islam et al., 2023; Karthikeyan, et al., 2022; La Rosa et al., 2022; Li et al., 2022; Reynolds et al., 2022; Róka et al., 2022; Vo et al., 2022; Wurtzer et al., 2022). Two approaches are commonly employed for variant detection: PCR-based methods such as RT-qPCR and RT-dPCR, and next-generation sequencing (NGS) followed by bioinformatics analysis. NGS provides high-throughput sequencing that can detect rare viral variants and provide detailed genomic characterization. However, implementing NGS requires significant expertise, infrastructure, and investment due to its high costs and complex bioinformatics analysis. On the other hand, the use of mutation RT-dPCR assays on SARS-CoV-2 RNA from wastewater can quickly, effectively and reliably monitor variants that are introduced and spread within a community (Tiwari et al., 2023). Due to these attributes, RT-dPCR was selected to track the BA.2.86 lineage that has surfaced in wastewater in recent months (Vigil et al., 2024).

In Italy, the BA.2.86 variant was initially reported in September 2023, appearing in two regions (Lombardia and Veneto), and accounting for 0.3% of the circulating variants according to clinical surveillance data (https://www.epicentro.iss.it/coronavirus/pdf/sars-cov-2-monitoraggio-varianti-indagini-rapide-settembre-2023.pdf). Subsequently, its prevalence escalated rapidly, reaching 1.3% in October, 11% in November, 51% in December and ultimately 85% in January (https://www.epicentro.iss.it/coronavirus/sars-cov-2-monitoraggio-varianti-indagini-rapide). The BA.2.86* showed a parallel evolutionary trend in wastewater samples. Its prevalence was very low in October, slightly increased in November, and had a significant surge in circulation in December and January. In particular, it is noteworthy that the sub-lineage JN.1 appeared in December 2023 and quickly replaced the parental BA.2.86 variant. In December, JN.1 had indeed a prevalence of 38.1% compared to 13.2% of BA.2.86. In January 2024, this trend intensified, with JN.1 dominating over BA.2.86 with 77.0% compared to only 6.1%.

During the four-month analysis, ISS conducted regular 'flash surveys' to sequence the long fragment (approximately 1400 bps) of the SARS-CoV-2 spike protein in wastewater samples using a previously established assay available on the ISS website (https://www.iss.it/cov19-acque-reflue). Sanger sequencing was employed, which is known to be less effective in identifying minor variants within complex matrices like wastewater, as it typically reveals prevalent ones. Indeed, while the BA.2.86* was detected in wastewater samples as early as October 2023 using the newly designed RT-dPCR method, it remained undetected by Sanger sequencing until December 2023, coinciding with the diffusion of the JN.1 sub-lineage across Italy. This suggests that the BA.2.86 lineage present in October and November, during periods of low variant circulation, escaped detection via Sanger sequencing due to its overshadowing by other prevalent variants (XBB.1.5*/XBB.1.9*, XBB.1.16*, XBB.2.3*, CH.1.1* and CM.7* variants). In December 2023, instead, 30% of sequences were identified as JN.1 by Sanger sequencing, a proportion that increased to 79% by January 2024. During December 2023 and January 2024, the JN.1 variant was detected at significantly higher proportion using both RT-dPCR and Sanger sequencing methodologies.

The here presented and used RT-dPCR assay accurately described the spread of the BA.2.86 lineage. However, unlike Sanger, this assay cannot differentiate sub-variants such as JN.1, but it only identifies the BA.2.86*. To confirm the specificity of the assay, and to attempt to discriminate JN.1, we sequenced by Sanger sequencing the short fragment amplified with the primers of the digital PCR, as JN.1 contains a specific mutation within this fragment, namely mutation L455S, not present in the other BA.2.86 sub-variants. All sequenced samples were identified as the JN.1 variant due to the presence of the L455S mutation. This confirms that the test can be used in combination with sequencing to differentiate between BA.2.86 and JN.1, which cannot be achieved using the RT-dPCR test alone.

Other studies monitored the spread of the BA.2.86 lineage through wastewater. In Sweden, the BA.2.86* was first detected in wastewater during the first week of August through NGS sequencing. It rapidly spread in the following weeks to most regions (Espinosa-Gongora et al., 2023). Genomic monitoring of SARS-CoV-2 in Berlin’s wastewater identified JN.1 as early as September 2023 using an Illumina COVIDSeq Test (Bartel et al., 2024). Results from wastewater surveillance sequencing in Denmark, indicated that the BA.2.86 variant was circulating in the country at a low level in mid-August (Rasmussen et al., 2023). The BA.2.86 variant was also detected in wastewater samples in Thailand as early as July 28, 2023 (Wannigama et al., 2023). Additionally, Wurtzer et al. used quantitative screening of BA.2.86 through RT-dPCR as part of wastewater monitoring program in France. Positive wastewater samples were first detected on 22 August 2023. Analysis of wastewater samples showed little circulation of this variant until 24 September. After that, a rapid acceleration was observed, leading to the detection of this variant in most wastewater in November (Wurtzer et al., 2024). NGS sequencing is usually preferred in most studies. However, a quantitative assay can also be a viable alternative, as it is an easily implemented, inexpensive, and highly informative approach to estimate the dynamics of emerging variants within populations.

The study has some limitations due to the low coverage of certain Italian regions, caused by the voluntary participation of SARI network laboratories in periodic flash surveys. This is reflected in the low number of samples analysed in certain areas and in the lack of consistency in sample collection, which could lead to bias in the representation of variant prevalence and circulation.

Overall, this study emphasises the importance of environmental surveillance as a tool for monitoring the emergence and spread of new variants, especially during periods of low virus circulation. Quantitative methods, such as RT-dPCR, can successfully detect single variant-specific mutations, increasing specificity and depth of detection in an environmental sample with multiple variants. The method presented provides a specific, sensitive and rapid approach to detect and monitor the presence of BA.2.86X variant in wastewater.

## Supplementary Information

Below is the link to the electronic supplementary material.

Supplementary file1 (XLSX 56 KB)

## Data Availability

No datasets were generated or analysed during the current study.
